# Developmental characteristics of orientation and differentiation abilities in the control of jumping distance in preschool children

**DOI:** 10.3389/fped.2025.1365323

**Published:** 2025-05-20

**Authors:** Hirohisa Kano, Alexander Kuga

**Affiliations:** ^1^ School of Health and Sport Sciences, Chukyo University, Toyota, Japan; ^2^ School of Education and Welfare, Aichi Prefectural University, Nagakute, Japan

**Keywords:** coordination ability, motor development, motor imagery, feedback, motor assessment

## Abstract

**Introduction:**

The development of coordination abilities and fundamental motor skills in early childhood plays a crucial role in promoting physical activity and preventing obesity. However, only a few studies have investigated the developmental characteristics of coordination abilities during early childhood. Therefore, we used jumping distance control as a motor task to examine the developmental characteristics of orientation and differentiation abilities in early childhood.

**Methods:**

We included 318 children aged 3.5–6 years. The motor task was a box target jump test in which the participants jumped from a box approximately 30 cm in height, such that their heels were aligned with a target line 40 cm away. Each participant performed the test two times. The performance results from the two box jump test trials were analyzed by comparing the mean errors of the first and second trials, along with a more detailed examination through the analysis of the performance level ratios between the two trials.

**Results:**

While the participants aged 3.5 years displayed insufficient accuracy and a strong tendency toward underdevelopment (low-performance percentage for 3.5 years: 1st = 38.3%, 2nd = 38.3%, *p* < 0.001), immediate feedback effects began to appear at the age of 4 years (low-performance percentage for 4 years: 1st = 21.3%, 2nd = 14.9%). Behavioral changes, such as improved accuracy between trials, became more evident from 4.5 years of age. These results suggest an emerging capacity for motor adjustment or imagery, although no direct assessment of motor imagery was conducted in this study, and such interpretations remain speculative. By approximately 5 years of age, participants obtained a certain level of immediate feedback effect.

**Conclusion:**

These findings provide insight into the developmental characteristics of coordination abilities in early childhood and could help inform age-appropriate physical education approaches that support movement awareness and adaptive motor control.

## 1 Introduction

The recent increase in childhood obesity, attributed to a decrease in physical activity (1, 2), has led to a decline in motor skills among children, emerging as a global issue. Children in the preschool age range develop their motor skills through physical activity (3–5). However, many do not meet the physical activity recommendations outlined in various national guidelines (6).

The impact of exercise experiences during early childhood on future exercise habits, physical activity levels, and health conditions, including obesity (7, 8), should not be disregarded. In this respect, reduced coordination and fundamental motor skills have been recognized as psychological barriers to physical activity, resulting in a detrimental cycle of obesity that is exacerbated by physical inactivity and leads to impaired mobility (9–11). Early childhood development must include adequate development of coordination ability and basic motor skills to promote physical activity and prevent obesity. However, basic coordination and motor skills have declined during early childhood, and current research on the developmental traits of coordination skills, especially during this stage, is inadequate. For example, studies have been conducted under limited conditions (e.g., focusing on specific coordination abilities and movements) (12–14) to identify age-specific characteristics. Therefore, gathering a wide range of studies that cover various disciplines and characteristics of movement is crucial for future research.

Coordination ability is defined as “organizing movements and the sensorimotor processes that underlie them toward a goal or purpose” (15). As a prerequisite for athletic performance, it serves as an essential ability to effectively conduct exercise or sports (16–18). Coordination ability can be structured into the following seven main abilities: balance, orientation, differentiation, reaction, rhythm, coupling, and changeover ability (19). Among these, the particularly important abilities are orientation, which accurately controls space and time, and differentiation, which adjusts muscle output for accurate movement (20). These two abilities are exerted through a series of judgment and execution movements. Exercise situations encountered in early childhood typically involve controlling one's body with respect to a target person or object, such as tags, ball games, or rope games. Orientation and differentiation play important roles in these activities; however, the developmental characteristics of these abilities have not been elucidated.

Previous studies, including ours, have examined the developmental characteristics of coordination abilities in early childhood, particularly focusing on orientation and differentiation in tasks such as throwing and jumping (12–14). These studies have suggested that the average performance in jumping tasks related to these abilities stabilizes at approximately 4.5 years of age, with a marked improvement occurring by 5 years of age. Additionally, girls tend to show earlier development than boys (12).

However, these findings were primarily based on evaluations using mean performance values across multiple trials, capturing the developmental characteristics of coordination abilities from a limited perspective. As such, these findings cannot be said to fully reflect the developmental patterns of coordination abilities in early childhood. Therefore, developing more detailed assessment methods that go beyond average values and account for performance differences between individual trials, considering changes from trial to trial is essential.

Germany, a leading nation in coordination research, has developed several test batteries to measure coordination ability, including the Körperkoordinationstest Für Kinder (21, 22) and MOT4-6 (23, 24) tests. Specifically, the Körperkoordinationstest Für Kinder test, designed to assess coordination ability, primarily targets balance and movement functions, such as walking backward, jumping sideways, and shifting platforms (25). However, these items primarily measure the speed of movement and do not specifically target orientation or differentiation skills, which are crucial in early childhood. Furthermore, Hirtz, a pioneer in coordination research among children, introduced ability-specific measurement items that specialize in coordination abilities, such as orientation, differentiation, and reaction (20, 26, 27). However, he and other colleagues focused mainly on the primary school years, with only a few ability-specific measurement items for young children. Furthermore, they did not describe the development characteristics of the children or the analysis methods that could be applied to the measurement results. It has also been pointed out that the coordination ability tests used in previous studies conducted in Germany may be biased toward specific types of movement and may insufficiently evaluate jumping movements.

Jumping movements are considered a fundamental locomotor skill in early childhood (28) and are among the primary motor movements that children must acquire and develop (29). Furthermore, various types of jump movements exist, such as jumping a long distance with maximum force, jumping to a specific location, and jumping over obstacles. This suggests that orientation ability, which dictates distance, and differentiation ability, which regulates jumping distance through muscle adjustments (14, 20), are movements observed in practical exercise scenarios and warrant consideration.

Compared to skills that have reached a certain level of automation, coordination ability is demonstrated through performance while attempting to adjust movements in each trial. Therefore, measurements and assessments of coordination ability should be divided into two rounds: an initial round, where the movement is created based on imagery (internally simulating future motor behavior without direct motor output) alone after facing the motor task for the first time, and a second round, where readjustments to the movement are made after obtaining feedback from the initial round (13, 30). Furthermore, considering the bias between the first and second performance levels is important because concrete differences in performance may be overlooked by comparing averages alone.

The current study sought to supplement previous research by examining individual trial performance. This approach enables the identification of age-related differences between the initial performance, where movements are created solely based on imagery, and the second performance, where movements are readjusted based on feedback from the first trial. Additionally, this method enabled a comprehensive analysis of the developmental traits associated with orientation and differentiation skills in jumping movements during the early stages of childhood. As such, the present study focused on jumping movements and investigated the developmental traits of orientation and differentiation abilities in young children, with a focus on jumping distance regulation. This study aimed to comprehensively assess developmental characteristics by comparing the performance level ratios between the first and second test trials, specifically using an evaluation method that incorporates mean values, as previously used in studies (12, 20, 26).

## 2 Materials and methods

### 2.1 Participants

Data used in this study were based on 318 preschool children (165 boys and 153 girls) aged 3.5–6 years who attended kindergartens in Aichi Prefecture, Japan, between 2018 and 2019. They were classified into six age groups in 6-month increments as follows: 3.5y, older 3-year-olds; 4y, younger 4-year-olds; 4.5y, older 4-year-olds; 5y, younger 5-year-olds; 5.5y, older 5-year-olds; and 6y, younger 6-year-olds. To contextualize the developmental stages of each age group, we briefly describe the general characteristics of gross and fine motor development between the ages of 3.5 and 6 years. During this period, children exhibit rapid improvements in postural control, balance, and body coordination, as well as increased precision in fine motor skills, such as hand manipulation and object control (28, 31). Sensory integration and the acquisition of body schema also advanced during these years, contributing to the development of motor planning and execution. These developmental changes provide a theoretical basis for understanding age-related differences in motor coordination tasks used in this study. *a priori* sample size calculation based on a repeated-measures analysis of variance within-between interaction design indicated that 324 individuals were required to detect a minimal effect size of f = 0.14 (alpha = 0.05, and 1-B = 0.95). Children with developmental delays were excluded. Table 1 presents the physical characteristics of the children. This study's purpose was explained in writing to the parents of the participating children in advance, and only those who provided consent were allowed to participate. The children's willingness to participate was confirmed before the implementation of the study. Approval for this study's protocol was obtained from the Research Ethics Review Committee of Aichi Prefectural University (Approval No.: 30 Aikendai-gakujo No. 11-2).

**Table 1:** Physical characteristics of children.

Age category	Height (cm)						Weight (kg)		
	Males		Females		Overall		Males	Females	Overall
	*n*	Mean	*n*	Mean	*n*	Mean	Mean	Mean	Mean
		(SD)		(SD)		(SD)	(SD)	(SD)	(SD)
3.5y	24	96.9	23	96.6	47	96.8	14.7	14.9	14.9
		(4.2)		(3.3)		(3.8)	(1.6)	(1.5)	(1.5)
4y	23	102.1	24	101.1	47	101.6	16.0	16.3	16.3
		(4.5)		(3.6)		(3.8)	(1.6)	(1.5)	(1.5)
4.5y	38	103.7	23	103.6	61	103.6	16.7	16.7	16.7
		(4.2)		(4.2)		(4.2)	(2.2)	(1.9)	(1.9)
5y	32	108.0	36	106.4	68	107.2	17.7	17.8	17.8
		(3.9)		(4.0)		(4.0)	(2.2)	(2.0)	(2.0)
5.5y	26	109.7	26	110.5	52	110.1	18.5	18.8	18.8
		(4.0)		(4.3)		(4.1)	(2.9)	(2.5)	(2.5)
6y	22	112.1	21	111.0	43	111.6	18.8	19.4	19.4
		(4.5)		(4.9)		(4.7)	(2.2)	(2.5)	(2.5)

SD, standard deviation; 3.5y, older 3-year-olds; 4y, younger 4-year-olds; 4.5y, older 4-year-olds; 5y, younger 5-year-olds; 5.5y, older 5-year-olds; 6y, younger 6-year-olds.

### 2.2 Content of motor tasks

**Figure 1:**
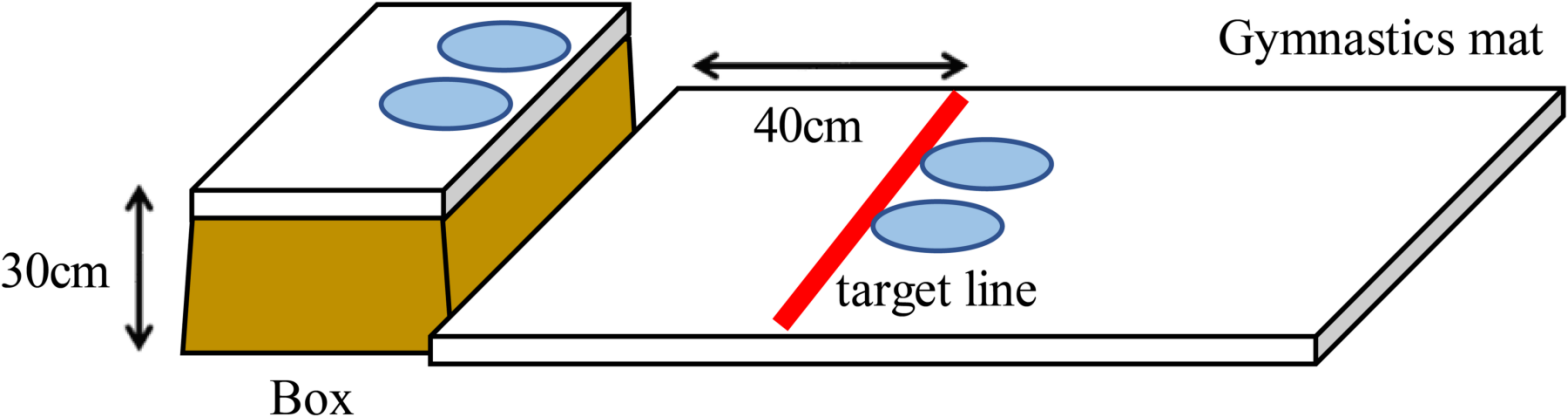
Box target jump test structure.

The jumping task involved a box target jump test, in which participants jumped from a box with a height of approximately 30 cm, such that their heels were aligned with a target line 40 cm away (Figure 1). According to previous studies, measurements were conducted two times in succession (12, 20, 26). The criteria used to evaluate the highest and lowest performance were a jump within a margin of error of up to 3 cm and one exceeding 12 cm, respectively. This test was used to measure the orientation capacity necessary for the spatial grasp of the jumping distance to the target line and the differentiation capacity necessary for the coordination of the muscle output of the jumping motion. A tape was used to measure the error from the target line to the heel. If the participant landed on their buttocks or was deemed to be unfocused, they had to start over. However, the distance from the target line to the farthest heel was measured if their feet were not aligned in the forward or backward direction when landing. If the foot position shifted after landing, the position where the participant first landed was used as the measurement value.

Measurements were conducted by a physical education instructor and a student who had received training in children's motor abilities. During the implementation process, the assessors demonstrated techniques to help the participating children understand. This included using footprints as feedback to indicate landing points before each trial.

### 2.3 Statistical analysis

To capture developmental trends by sex and age in the box target jump test, a repeated-measures three-factor analysis of variance was conducted with sex and age as between-participant factors and between-trial (first and second trials) as within-participant factors. The absolute error from the target line in each trial was used as the dependent variable. When interactions were significant, a simple main-effect test was conducted using the Bonferroni method. Next, errors from the target line were classified into three levels based on previous research to capture the features of the age groups in the first and second trials (20, 26). The following classifications were used: jumping with an error of up to 3 cm, which was assessed as the highest performance (“high performance”); jumping with an error exceeding 12 cm, which was assessed as the lowest performance (“low performance”); and jumping with an error that falls between these two performances (“middle performance”). We conducted a cross-tabulation (frequency and proportion) between these three levels and each age group, a chi-square test to test independence, and Cramer's *V*-test to measure the effect size. If the chi-square analysis indicated non-independence, a residual analysis was conducted, and cells with an adjusted standardized residual > |1.96| were considered statistically significant (32). The following effect size was assessed as follows: small, ≥0.1; medium, ≥0.3; and large, ≥0.5 (33, 34). All statistical analyses were performed using the SPSS statistical package PASW Statistics 28 (IBM Corp., Armonk, NY, USA). The statistical significance level of the results in this study was set at 5% for all cases.

## 3 Results

### 3.1 Physical characteristics

Table 1 presents the physical characteristics of the children according to sex and age. The results indicated that both height and weight increased with age. Additionally, the results of a *t*-test by age to confirm sex differences showed no significant differences in height or weight.

### 3.2 Analysis by means of errors

**Figure 2:**
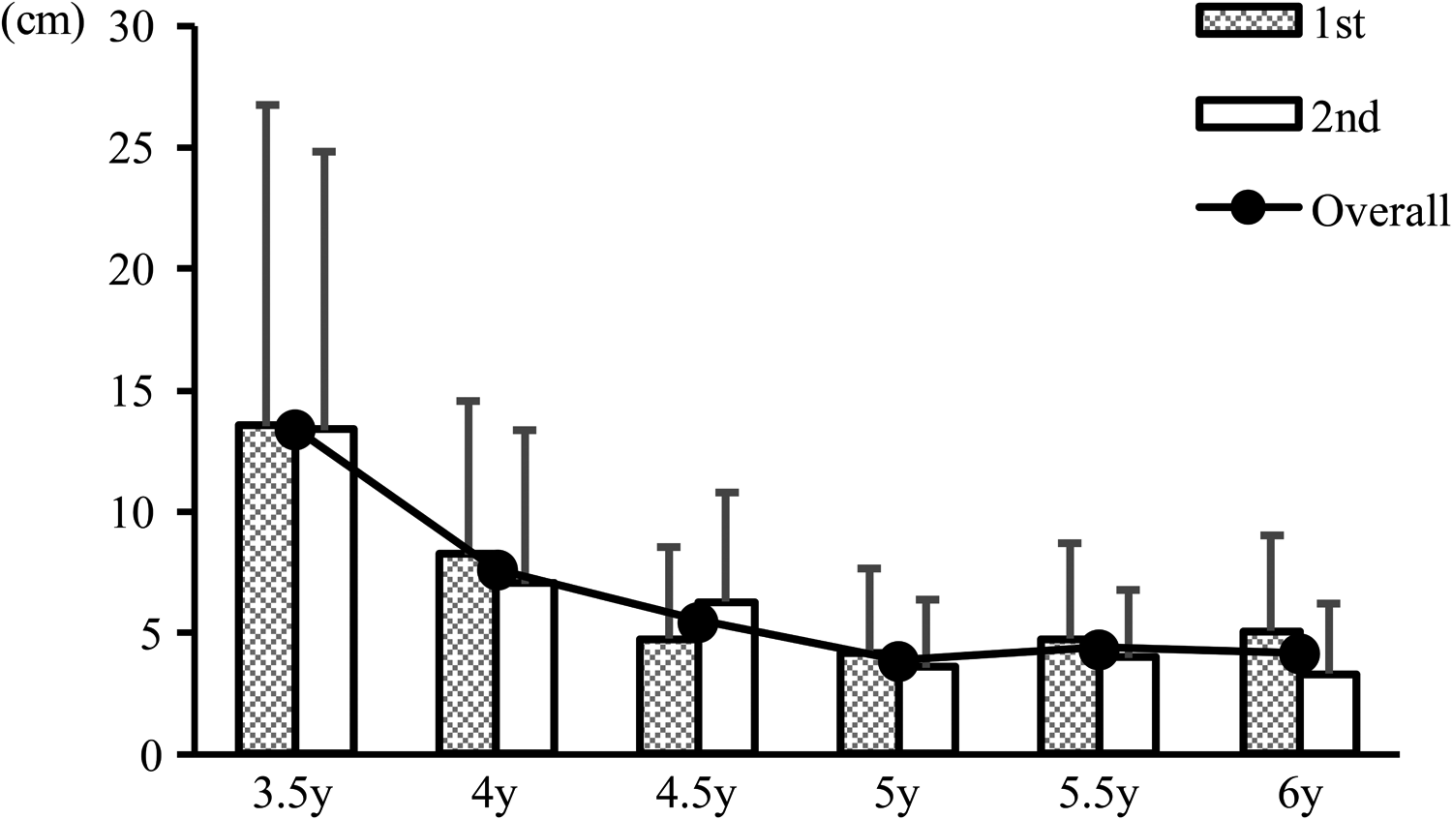
Change in mean value by age category. 3.5y, older 3-year-olds; 4y, younger 4-year-olds; 4.5y, older 4-year-olds; 5y, younger 5-year-olds; 5.5y, older 5-year-olds; 6y, younger 6-year-olds; 1st, first test; 2nd, second test.

Table 2 shows the mean error values for the box target jump, Table 3 presents the three-way analysis of variance results, and Figure 2 shows the changes with age. The three-way analysis of variance results showed that a main effect was observed only for the between-participants factor of age. Multiple comparison test results showed that the mean errors from 4 to 5 years onwards were significantly lower than those at 3.5 and 4 years, respectively. No other sex differences or inter-trial differences were observed, and no interactions were observed.

**Table 2:** Average of box target jump test.

Age category	Males		Females		Overall	
	1st	2nd	1st	2nd	1st	2nd
	Mean	Mean	Mean	Mean	Mean	Mean
	(SD)	(SD)	(SD)	(SD)	(SD)	(SD)
3.5y	14.56	14.88	12.43	11.78	13.52	13.36
	(15.0)	(13.2)	(11.4)	(9.4)	(13.2)	(11.5)
4y	9.09	6.22	7.40	7.83	8.22	7.04
	(7.0)	(6.0)	(5.5)	(6.5)	(6.3)	(6.3)
4.5y	4.70	5.68	4.78	7.17	4.73	6.25
	(3.9)	(4.2)	(3.7)	(5.0)	(3.8)	(4.6)
5y	4.42	3.92	3.89	3.29	4.14	3.59
	(3.6)	(3.1)	(3.4)	(2.4)	(3.5)	(2.8)
5.5y	4.37	4.08	5.13	3.90	4.75	3.99
	(4.1)	(3.2)	(3.9)	(2.4)	(4.0)	(2.8)
6y	5.66	3.50	4.43	3.07	5.06	3.29
	(3.1)	(3.2)			(2.9)	

SD, standard deviation; 3.5y, older 3-year-olds; 4y, younger 4-year-olds; 4.5y, older 4-year-olds; 5y, younger 5-year-olds; 5.5y, older 5-year-olds; 6y, younger 6-year-olds.

**Table 3:** Results of three-way ANOVA (sex, age, and between tests).

Source		df	F	*p*	ηp^2^	Multiple comparison
Between-participant factors	Sex	1	0.67	.413	.002	3.5y < 4y, 4.5y, 5y, 5.5y, 6y 4y < 5y, 5.5y, 6y
	Age	5	23.37	<.001*	.276	
	Sex × age	5	0.63	.680	.010	
Within-participant factors	Between tests	1	1.68	.196	.005	
	Sex × Between tests	1	0.68	.411	.002	
	Age × Between tests	5	1.92	.092	.030	
	Sex × age × Between tests	5	0.84	.520	.014	

3.5y, older 3-year-olds; 4y, younger 4-year-olds; 4.5y, older 4-year-olds; 5y, younger 5-year-olds; 5.5y, older 5-year-olds; 6y, younger 6-year-olds; ANOVA, analysis of variance.

* *p* < 0.05.

### 3.3 Ratio of performance levels for each test

The characteristics of the age groups in the first and second trials were determined by classifying the performance into three levels and conducting cross-tabulation with age groups, as well as chi-square tests (effect size: Cramer's *V*) (Table 4). A significant difference was found in both the first and second trials (*p* < 0.001), and a moderate effect was observed (1st trial: Cramer's *V* = 0.30; 2nd trial: Cramer's *V* = 0.36). A residual analysis results showed that, for both the first and second trials, participants aged 3.5 years had a significantly lower and higher number of high and low performances, respectively. In the first trial, participants aged 4 years had a significantly lower and higher number of high and low performances, respectively. However, no significant differences were observed in the second trial. Participants aged 4.5 years had a significantly lower number of low performances in the first trial, a significantly lower number of high performances, and a significantly higher number of middle performances in the second trial. Those aged 5 years had a significantly lower number of low performances in the first trial, a significantly higher number of high performances, and a significantly lower number of middle and low performances in the second trial. In particular, no participants were in the low-performance category. During the initial trial, no significant variation was observed among participants aged 5.5 years in any of the three performance levels. However, a significant decrease in performance was observed during the second trial since none of the participants met the criteria. Among the participants aged 6 years, a marked decrease in performance was observed during the initial trial. A notable increase in high performance and a significant decrease in low performance was observed during the second trial, with no participants achieving results.

**Table 4:** Cross-tabulation and chi-square test with age group.

Age category		1st					2nd				
		High	Middle	Low			High	Middle	Low		
	*n*	*n*	*n*	*n*			*n*	*n*	*n*		
		(%)	(%)	(%)	*p*	ES	(%)	(%)	(%)	*p*	ES
3.5y	47	**12**	17	**18**	<.001*	0.30	**9**	20	**18**	<.001*	0.36
		**(25.5)**	(36.2)	**(38.3)**			**(19.1)**	(42.6)	**(38.3)**		
4y	47	**12**	25	**10**			16	24	7		
		**(25.5)**	(53.2)	**(21.3)**			(34.0)	(51.1)	(14.9)		
4.5y	61	30	29	**2**			**20**	**35**	6		
		(49.2)	(47.5)	**(3.3)**			**(32.8)**	**(57.4)**	(9.8)		
5y	68	34	33	**1**			**44**	**24**	**0**		
		(50.0)	(48.5)	**(1.5)**			**(64.7)**	**(35.3)**	**(0.0)**		
5.5y	52	26	22	4			24	28	**0**		
		(50.0)	(42.3)	(7.7)			(46.2)	(53.8)	**(0.0)**		
6y	43	19	23	**1**			28	15	**0**		
		(44.2)	(53.5)	**(2.3)**			(65.1)	(34.9)	**(0.0)**		

ES, effect size; 3.5y, older 3-year-olds; 4y, younger 4-year-olds; 4.5y, older 4-year-olds; 5y, younger 5-year-olds; 5.5y, older 5-year-olds; 6y, younger 6-year-olds.

Adjusted standardized residuals greater than 1.96 are shown in bold, and those less than −1.96 are shown in bold and hatched.

* *p* < 0.05.

## 4 Discussion

We used jumping distance control as a motor task to examine the developmental characteristics of orientation and differentiation abilities in early childhood. The performance results of the box target jump test, which is a motor task, were analyzed from two perspectives. The first perspective involved analyzing the mean errors in the first and second trials according to previous research (12, 20, 26), whereas the second involved analyzing the performance level ratios of the two trials. Notably, the results revealed the following developmental characteristics of orientation and differentiation abilities in early childhood. Participants aged 3.5 and 4 years tended to be underdeveloped but began to show an immediate feedback effect, although the accuracy was insufficient in participants aged 4 years compared with those aged 3.5 years. This particular period is widely regarded as a significant turning point in the field of development studies (35). Although temporary stagnation in development is believed to have impeded the immediate feedback effect, speculation remains. The emergence of language regulatory abilities indicates that at approximately 5 years of age, children demonstrate greater proficiency in body control and coordination (12). Several factors, including the influence of verbal instructions from the measurer in each trial, can be considered. However, a certain degree of immediate feedback can be obtained during this timeframe.

Within the realm of motor learning, movements are refined and automated through the process of detecting and correcting errors repeatedly, as evidenced by previous studies (36, 37). However, during the initial and subsequent trials, the research demonstrated an estimated age at which immediate coordination of movement and improved performance were achieved—specifically, the acquisition of orientation and differentiation skills in jumping were attained. As far as we know, no such study has been conducted so far. In practice, research on children's jumping movements has focused primarily on standing long (38–40) or vertical (29, 41, 42) jumps, measuring and evaluating the maximum effort exerted during exercise. As demonstrated here, motor skills encompass not only physical attributes such as muscle strength and explosive force but also coordination abilities governed by the nervous system, which directs bodily movements toward specific objectives (43). During the early stages of childhood, a significant development of the nervous system occurs after birth (44). By 3 years of age, the number of neurons in the brain is equivalent to that of an adult, which forms the foundation of neural circuits (45). Therefore, elucidating the developmental characteristics of coordination abilities during early childhood, when the nervous system is significantly developed, is imperative for motor development research.

The use of the mean error in the analysis indicated a notable improvement in performance between 3.5 and 4.5 years without subsequent significant alterations. Additionally, orientation and differentiation skills appeared to reach a plateau at approximately 4.5 years of age. It can be deduced that a ceiling effect may be observed during motor tasks. However, previous research on throwing and jumping movements has also shown that orientation and differentiation abilities plateau at approximately 4.5 years of age (12–14). As such, performing more challenging motor tasks is imperative to assess the advanced developmental stages beyond 4.5 years of age. Additionally, directing attention to the performance level ratios of the initial and subsequent trials revealed more comprehensive developmental characteristics.

Initially, when examining the performance level ratio in the initial trial, the findings can be categorized into two primary patterns between age groups of 3.5 and 4 years, as well as ≥4.5 years. The findings indicated that the participants aged 3.5 and 4 years had a significantly lower and higher number of high and low performances, respectively, than those aged ≥4.5 years. On the contrary, participants aged ≥4.5 years tended to exhibit a significantly lower number of low performances. The findings of this study were consistent with those of previous research (12–14) on mean error analysis. Children aged 3.5 and 4 years had underdeveloped orientation and differentiation abilities compared to those aged ≥4.5 years.

Furthermore, when focusing on the performance level ratio in the second trial, the 4-year-old participants did not show statistically significant differences, but three trends could be distinguished between those aged 3.5, 4.5, and 5 years. Participants aged 3.5 years had a significantly lower and higher number of high and low performances, respectively, as in the first trial. Those aged 4.5 years had a significantly lower and higher number of high and middle performances, respectively, and the variation was larger than that in the first trial. No instances of low performance were recorded after 5 years of age.

Therefore, the analyses of the mean error and performance level ratio in the first trial showed that participants aged 3.5 and 4 years tended to be underdeveloped, whereas those in the second trial showed the above-mentioned differences. Our findings show that the participants readjusted their movements in the second trial after receiving feedback from the first trial, suggesting that an immediate feedback effect began to occur; however, the accuracy was insufficient for participants aged 4 years compared to those aged 3.5 years.

Compared to participants aged 3.5 or 4 years, a significantly lower number of low performances were observed from the first trial stage for those aged 4.5 years, with this trend being maintained, except for participants aged 5.5 years, where no statistically significant differences were shown. It has been hypothesized that the initial trial may have induced movement solely through imagery, suggesting that motor imagery develops from 4.5 years of age and beyond. The development of motor imagery shows significant progress from early childhood to school age (46) and is evident by the age of 5 years (47). However, since this study did not include direct measurements or assessments of motor imagery, any reference to motor imagery must be cautiously interpreted. Our results of classifying and analyzing the children's ages at 6-month intervals suggested behavioral changes from 4.5 years that reflect the emergence of motor imagery, which has been reported to develop at approximately 5 years of age in previous studies. However, as motor imagery was not directly assessed in this study, this interpretation remains speculative and should be confirmed in future research using appropriate methodological approaches. These include the possible effects of natural development due to aging and the effects of movement experiences on development.

When focusing on the second trial, participants aged 4.5 years had a significantly higher and lower number of middle and high performances, respectively. Although no statistically significant difference was observed, the number of participants with low performance slightly increased. These results suggest that the accuracy of the feedback effect is low at 4.5 years of age. This trend at 4.5 years of age overlaps with the developmental process of motor learning (43), which follows a process of acquisition, improvement, and stabilization, with a temporary plateau in performance during the improvement stage before resuming improvement. The developmental characteristics of orientation and differentiation abilities in early childhood reached a plateau at approximately 4.5 years of age, which may have manifested as a decrease in the accuracy of the feedback effect. In the second trial (from 5 years onwards), the number of low performances became significantly lower for 0 people, and a significantly higher number of high performances were recorded in participants aged 5 and 6 years, excluding children aged 5.5 years for whom no statistically significant difference was observed. A certain level of immediate feedback effect was obtained at approximately 5 years of age.

Our study obtained more detailed developmental characteristics of orientation and differentiation abilities among children aged 3.5–6 years, which have only been broadly clarified in previous analyses that used mean error values by performing an analysis employing the performance level ratio by trial. Notably, this study has provided new findings in the field of children's motor development research, particularly in coordination research. However, it has some limitations. First, in the present study, no obvious differences were observed from 5 years onwards; therefore, discussing the developmental characteristics from 5 to 6 years of age was impossible. In the future, examining the developmental characteristics of orientation and differentiation abilities from 5 years of age onwards using motor tasks with differing difficulty levels will be necessary. Moreover, as this study employed a cross-sectional design, we must acknowledge that differences observed across age groups (e.g., between 4- and 5-year-olds) do not necessarily indicate developmental change over time. These differences may reflect a range of factors, such as prior movement experiences, parental involvement in physical play, or environmental conditions. Therefore, longitudinal research is needed to further explore the causal mechanisms and intra-individual changes underlying the development of coordination abilities. Second, the box target jumps used in this study are insufficient when used alone to measure orientation and differentiation abilities. Therefore, orientation and differentiation abilities should be examined from multiple perspectives by increasing the variation in motor tasks while assuming different levels of difficulty. Third, although this study referred to feedback effects and motor imagery in explaining performance improvement, it did not directly assess these processes. Future research should examine these cognitive mechanisms more rigorously, ideally using neuroscientific approaches. Furthermore, in light of the interrelation between motor skills and cognitive functions (48, 49), approaching the study of coordination abilities from a neuroscientific perspective to better understand its underlying mechanisms appears imperative.

## 5 Conclusion

This study clarified the developmental characteristics of orientation and differentiation abilities in children aged 3.5–6 years by examining changes in performance level ratios across repeated trials.

Our findings suggest that the ability to adjust movements based on feedback begins to emerge from approximately 4.5 years of age and becomes more accurate at 5 years of age. These results imply that movement tasks that incorporate opportunities for immediate feedback may be particularly effective in developing coordination abilities in early childhood. Furthermore, in early physical education settings, introducing playful movement experiences that promote trial-and-error and self-correction could support the development of coordination and body control, even before formal instruction becomes appropriate.

## Data Availability

The raw data supporting the conclusions of this article will be made available by the authors, without undue reservation.
